# Optimization of AIN Composite Structure Based Surface Acoustic Wave Device for Potential Sensing at Extremely High Temperature

**DOI:** 10.3390/s20154160

**Published:** 2020-07-27

**Authors:** Shuyao Fan, Wen Wang, Xueling Li, Yana Jia, Yuan Sun, Mengwei Liu

**Affiliations:** 1 Institute of Acoustics, Chinese Academy of Sciences, Beijing 100190, China; fanshuyao@mail.ioa.ac.cn (S.F.); lixueling@mail.ioa.ac.cn (X.L.); jiayana@mail.ioa.ac.cn (Y.J.); sunyuan@mail.ioa.ac.cn (Y.S.); liumw@mail.ioa.ac.cn (M.L.); 2 School of Electronic, Electrical and Communication Engineering, University of Chinese Academy of Sciences, Beijing 100190, China

**Keywords:** surface acoustic wave, high-temperature sensor, composite structure, finite element method, coupling of modes simulation

## Abstract

A surface acoustic wave (SAW) device with an aluminum nitride (AlN) composite structure of Al_2_O_3_/IDTs/AlN/Metal/Si was proposed for sensing at extreme high-temperature in this work. Optimization allowing determination of optimal design parameters for SAW devices was conducted using the typical coupling of modes (COM) model. The SAW propagation characteristics in the layered structure were investigated theoretically by employing the finite element method (FEM). Multiple acoustic-wave modes that occurred in the AlN composite structure was analyzed, and the corresponding suppression of spurious mode was proposed. The COM simulation parameters corresponding to the effective acoustic-wave mode were extracted, and the optimized parameters of the one–port SAW resonator with a high-quality factor were determined.

## 1. Introduction

High-temperature sensors are essential for obtaining the physical information of critical components at extremely high temperatures in fields such as aerospace, energy and chemical engineering, and nuclear power [1,2,3]. Among the currently available sensor technologies, surface acoustic wave (SAW) devices have attracted more attention due to the features of small size, low cost, fast response, strong anti-interference ability, and is wireless and passive [4,5]. The SAW was generated by sputtering metal interdigital transducers (IDTs) on top of the piezoelectric crystal. Clearly, to build the SAW chip for sensing at extremely high temperature, the piezoelectric crystal and metal electrode should withstand extreme high temperature.

Typical piezoelectric crystals are not suitable for sensing at extremely high temperatures. For example, when the applied temperature exceeds 350 °C, large acoustic attenuation will occur for the ST–Quartz. Additionally, the phase transition temperature of quartz is very low (573 °C), which limits its maximum operating temperature. The lithium niobate (LiNbO_3_) single crystal features high electromechanical coupling factor (K^2^), but its temperature stability is inferior. When the temperature exceeds 300 °C, its chemical composition will begin to decompose. The yttrium calcium oxyborate (YCOB), lanthanum gallium silicate (LGS), and aluminum nitride (AlN) have been explored for sensing at extremely high temperatures in recent years. These materials all have a high phase transition temperature and stable performance above 800 °C [6,7,8]. However, YCOB suffers from the pyroelectric effect, and its surface is easy to absorb impurities in the environment at high temperature, which causes severe degradation of device performance. LGS also has many restrictions such as low surface acoustic velocity and increasing acoustic propagation losses with the temperature at higher frequency [9]. Compared with the former two, aluminum nitride (AlN) exhibits higher surface wave velocity (5607 m/s) [10]. It is effortless to prepare this material by standard crystal growth methods [11]. Furthermore, the most important is that the fabrication of AlN based SAW devices can be compatible with conventional silicon technology [12]. On the other hand, platinum (Pt) and its alloys are widely known for its high thermal and chemical robustness, and are commonly used in micro-machined devices operated in an extreme environment like, for example, sensors and micro heaters [13,14]. Therefore, a combination of Pt and the AlN substrate provides an effective way of building a high-temperature sensor. Some cases of SAW device based on AlN composite structure employing Pt electrodes for sensing at high temperature have been reported in recent studies [11,15,16,17].

A high-performance sensing chip is a prerequisite for efficient sensor technology. Obvious acoustic wave attenuation and deterioration of Q–value will occur in a SAW sensing device at extremely high temperatures. Hence, when choosing high temperature-resistance piezoelectric substrates and metal electrodes, optimization of the SAW sensing device allowing larger Q-values and low attenuation are also very important. Unfortunately, corresponding works dealing with the AlN composite structure based SAW sensing device are rare.

In this work, a SAW device configuration with the multilayer structure of Al_2_O_3_/IDTs/AlN/metal thin-plate/Si was proposed for sensing physics at extremely high temperatures. The corresponding theoretical analysis on acoustic wave propagation in the layered structure was conducted by using the finite element method (FEM), and the multi–acoustic wave modes originating from the multi-reflection in composite structure and corresponding way of suppression was demonstrated. Then, the theoretical simulation for the AlN composite structure-based SAW device was performed by employing the conventional coupling of modes (COM) model, and optimal design parameters allowing larger Q–value were determined.

## 2. Propagation Characteristics of the surface Acoustic Wave (SAW) Sensor

**Figure 1:**
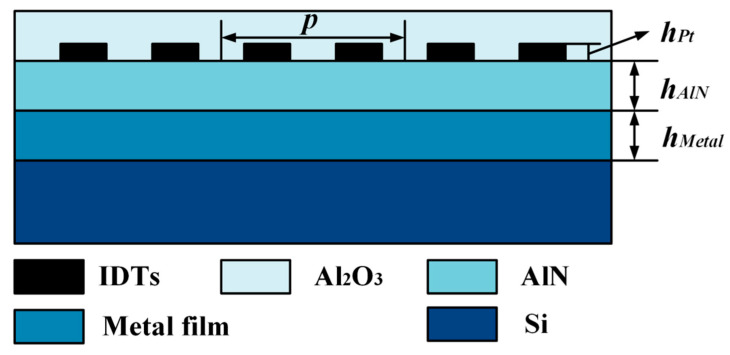
The cross-section schematic of the surface acoustic wave (SAW) device built by composted aluminum nitride (AlN) structure.

In this contribution, the excitation and propagation characteristics of SAW in the multilayer composite structure of Al_2_O_3_/IDTs/AlN/metal thin–plate/Si were investigated theoretically by the finite element (FEM) analysis. Figure 1 depicts the cross-section schematic of the SAW device built by composted AlN structure, where p, h_AlN_, h_Pt_, and h_Metal_ denote the period of IDTs, AlN thickness, Pt electrode thickness, and Pt thin–plate thickness, respectively.

**Figure 2:**
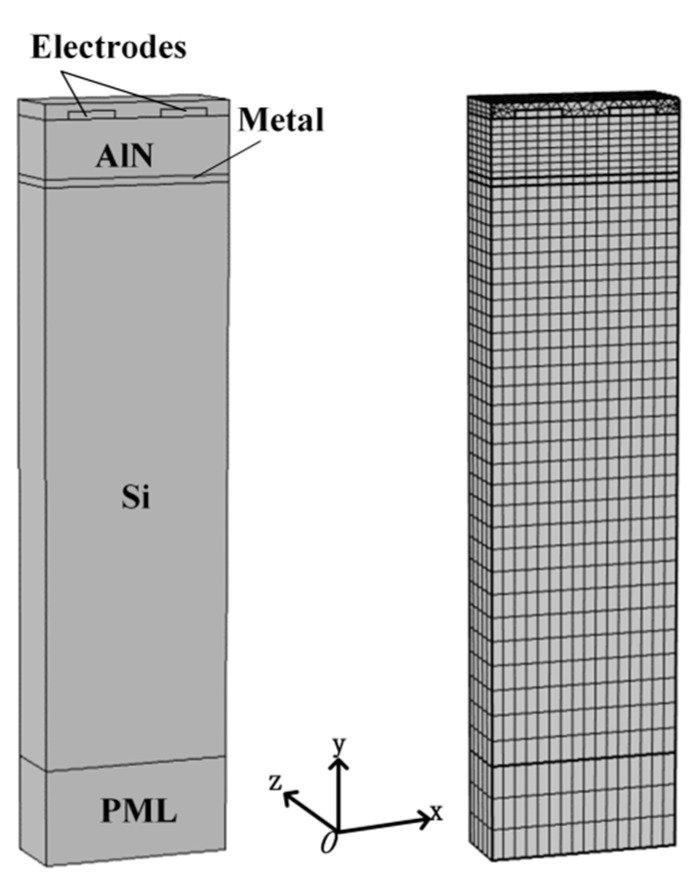
The three-dimensional (3D) periodic model of the proposed SAW device.

Figure 2 shows the three-dimensional (3D) periodic model of the proposed SAW device. The hypothesis of continuous periodicity was set to improve the computation efficiency in the computation. It is well-known that the acoustic energy is confined in the piezoelectric crystal surface within several wavelength depths. Hence, the perfect matching layer (PML) can be used to reduce the thickness of the Si substrate, which can save the calculation time and effectively suppress the spurious waves from the interface reflection [14]. The corresponding material constants are revealed in the series literature [18,19,20,21].

The modal analysis was performed on the proposed SAW periodic structure to extract the possible characteristic frequency. Then, the appropriate frequency scanning range was chosen around the obtained natural rate. Next, the input admittance (Y) within a certain frequency can be obtained by applying the excitation electrical signal to the IDT. Furthermore, the edge frequency can be derived from the input admittance for infinitely-long IDT, where f_sc+_, f_sc−_, f_oc+_, and f_oc−_ denote the lower and upper boundary frequencies of the stopband in the periodic short-circuit and open-circuit gratings, respectively. Finally, the electrostatic field energy can be gained by the static analysis.

There are many acoustic modes in the multi-layer composite structure, but devices developed based on different modes exhibit their own advantages. For example, the high acoustic velocity mode helps to create high-frequency SAW devices. Additionally, some acoustic wave modes reveal a larger K^2^, benefiting reducing insertion loss and improvement of the Q-factor. However, the presence of spurious modes will occupy acoustic energy, increasing acoustic attenuation of the primary acoustic wave mode, and the Q–value of the corresponding device will also be decreased. This means that the baseline noise will be increased, which will further affect the wireless sensing performance. Therefore, we hope to optimize the different acoustic wave modes and suppress the corresponding spurious modes by analyzing the SAW propagation, and a high-Q and low loss SAW sensor could be realized.

**Figure 3:**
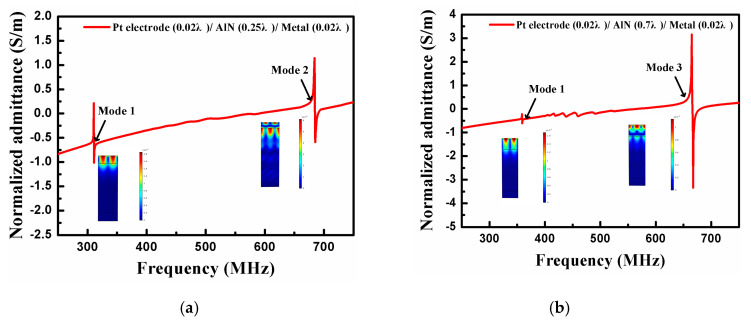
The input admittance when (**a**) AlN film thickness h_AlN_ = 0.25 λ and (**b**) AlN film thickness h_AlN_ = 0.7 λ.

Figure 3 indicates the relationship between frequency f and input admittance Y in the infinite IDT. As shown in Figure 3a, when the h_AlN_ is 0.25 λ (λ is the acoustic wavelength), two distinct SAW modes can be observed in the calculation. In this example, mode 1 represents the Rayleigh wave mode, and the resonance frequency is about 310 MHz. Meanwhile, mode 2 is an A-type Lamb wave mode [19,22,23,24], with the resonance peak position at about 690 MHz. Additionally, a new acoustic mode emerges at a fixed h_AlN_ of 0.7 λ, as shown in Figure 3b. Here, mode 3 is the S-type Lamb wave mode [19,22,23,24], and it dominates at ~670 MHz. 

Quite evidently, the presence of multi-acoustic waves will degrade the device performance, so the expression on spurious wave modes other than the defined wave mode should be conducted. Significantly, the acoustic modes in the device are continually changing with the variation of AlN thickness. These modes may be generated or suppressed. Consequently, it is feasible to achieve the excitation of main mode and suppression of spurious resonance by changing the device structure parameters.

According to COM theory, the expression of COM parameters can be obtained by Equations (1)–(3) [14]. Here, *v*, *κ_p_*, *α*, *C_n_*, *W*, and *W_e_* are defined as acoustic velocity, coupling coefficient, excitation coefficient, normalized static capacitance, acoustic aperture, and the electrostatic field energy, respectively.

(1) $$\{\begin{array}{l} v=\lambda \frac{\left(f_{\mathrm{sc}+}+f_{\mathrm{sc}-}\right)}{2},\\ \left|\kappa \right|=\frac{2\pi }{\lambda }\frac{f_{\mathrm{sc}+}-f_{\mathrm{sc}-}}{f_{\mathrm{sc}+}+f_{\mathrm{sc}-}}. \end{array}$$

(2) $$\{\begin{array}{l} \left|\alpha \right|=\sqrt{\frac{\omega C_{n}W\pi }{\lambda^{2}}\left(\frac{f_{\mathrm{oc}+}+f_{\mathrm{oc}-}}{f_{\mathrm{sc}+}+f_{\mathrm{sc}-}}-1\right)},\\ \cos\left(∠\alpha^{2}/\kappa \right)=\frac{{\left(f_{\mathrm{oc}+}-f_{\mathrm{oc}-}\right)}^{2}-{\left(f_{\mathrm{sc}+}-f_{\mathrm{sc}-}\right)}^{2}-{\left[\left(f_{\mathrm{oc}+}+f_{\mathrm{oc}-}\right)-\left(f_{\mathrm{sc}+}+f_{\mathrm{sc}-}\right)\right]}^{2}}{2\left(f_{\mathrm{sc}+}-f_{\mathrm{sc}-}\right)\left[\left(f_{\mathrm{oc}+}+f_{\mathrm{oc}-}\right)-\left(f_{\mathrm{sc}+}+f_{\mathrm{sc}-}\right)\right]}. \end{array}$$

(3) $$C_{n}=\frac{W_{e}}{{\left(\Delta V\right)}^{2}W}.$$

The resonance *f_r_* and anti–resonance frequencies *f_a_* can be developed from input admittance for each SAW mode, then corresponding effective K^2^ be estimated by Equation (4) [25].

(4) $$K^{2}=(\frac{\pi f_{r}}{2f_{a}})\cot(\frac{\pi f_{r}}{2f_{a}}).$$

**Figure 4:**
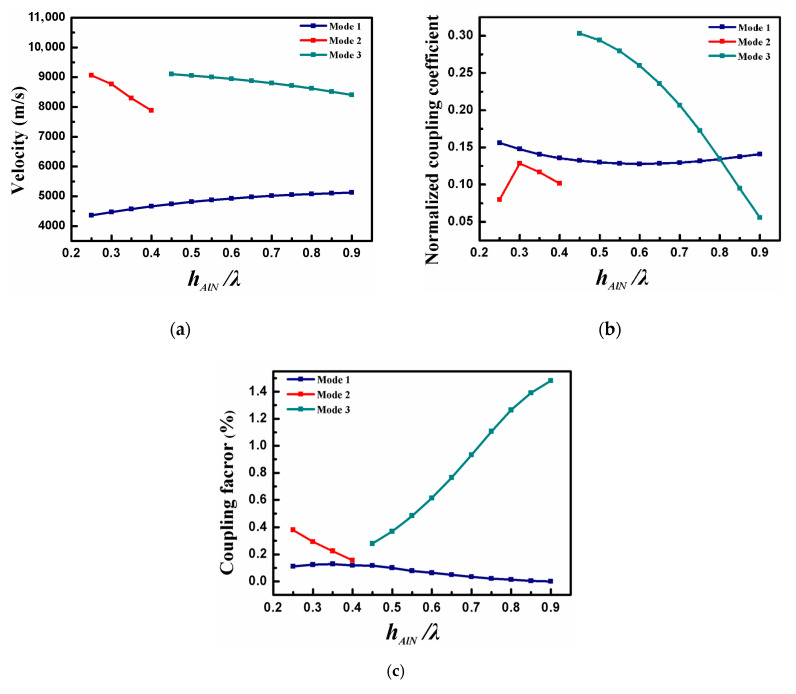
The calculated (**a**) v, (**b**) κ_p_, and (**c**) K^2^ as the function of h_AlN_/λ.

The Pt electrode thickness and the metal thin-plate thickness were both set to 0.02 λ. Figure 4 depicts the COM parameters as the function of normalized AlN thickness (h_AlN_/λ). It is seen that mode one and mode 2 are the main acoustic wave modes at a thin-AlN film. However, as the AlN thickness increases, mode one and mode 2 are gradually suppressed while mode three is generated. When the h_AlN_/λ is over 0.45, mode one and mode 3 become the main propagation modes, and mode 2 has almost disappeared.

It can be found that the acoustic velocity of each mode will gradually tend to the acoustic speed in AlN (Figure 4a), and more and more acoustic energy is concentrated in the piezoelectric material while h_AlN_/λ increases. As shown in Figure 4b, mode 1 takes a minimum value at h_AlN_/λ = 0.6, and the maximum κ_p_ of mode 2 can be observed when h_AlN_/λ is at 0.3. Furthermore, the reflection coefficient of mode 3 decreases with the increase in AlN thickness. We can see from Figure 4c that the K^2^ of mode 3 is much larger than mode 1 and mode 2. This work implies that the electric field generated by the IDT fits well with mode 3, and mode 3 is efficiently generated.

**Figure 5:**
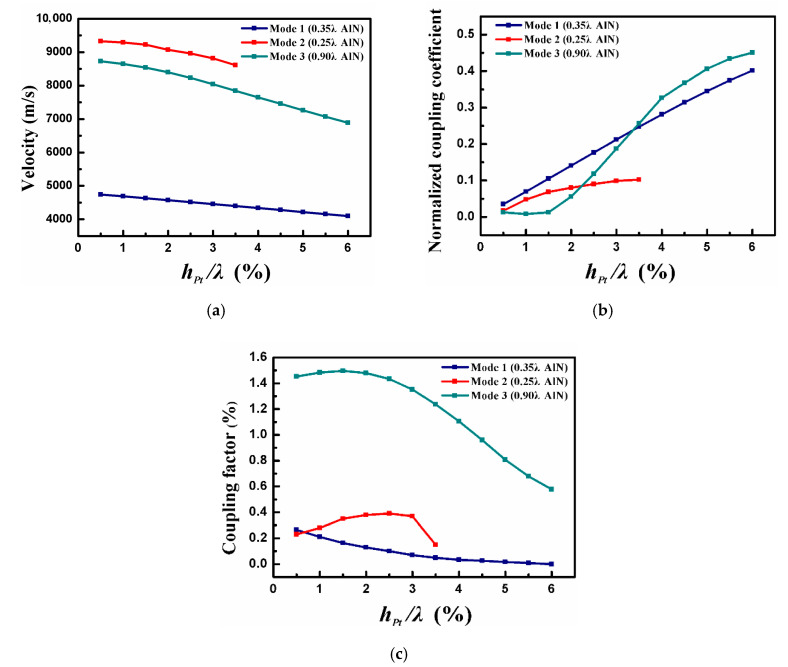
The calculated (**a**) v, (**b**) κ_p_, and (**c**) K^2^ as the function of h_Pt_/λ.

According to the variation trend of COM parameters, the appropriate AlN thickness corresponding to three acoustic modes is determined. Next, the propagation characteristics of the modes with different AlN thickness were analyzed critically for device optimization. The thickness of the metal thin-plate remained the same here. Figure 5 offers v, κ_p_, and K^2^ varying with h_Pt_/λ, where h_Pt_/λ denotes the normalized Pt electrode thickness.

As shown in Figure 5a, due to the mass loading, the acoustic wave velocity decreased monotonously with h_Pt_/λ for all modes. Whether it is mode 1, mode 2, or mode 3, the corresponding reflection coefficients first increased and then decreased as the Pt electrode thickness (Figure 5b), which was 2, the result of the interaction between the electrical load and the mechanical load. From Figure 5c, a maximum coupling coefficient of 0.27% for mode 1 was obtained when h_Pt_/λ is 0.005. Moreover, when h_Pt_/λ was selected as 0.03, a maximum value of K^2^ can be achieved for mode 2. Finally, a maximum K^2^ for mode 3 was obtained by 1.49% when the Pt electrode was set to 0.015 λ. Additionally, it is necessary to choose the appropriate electrode thickness because the SAW propagation loss increases with Pt electrode thickness.

**Figure 6:**
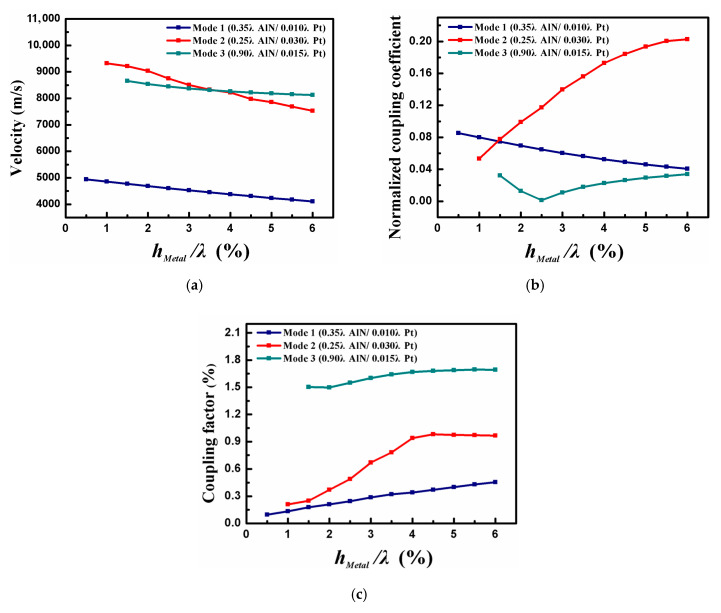
The calculated (**a**) v, (**b**) κ_p_, and (**c**) K^2^ as the function of h_Metal_/λ.

To analyze the SAW propagation characteristics in multilayer composite structure well, the influence of metal thin-plate thickness on device performance was also studied. Figure 6 reveals the relationship between v, κ_p_, and K^2^ with h_Metal_/λ, where h_Metal_/λ indicates the normalized metal thin-plate thickness. It can be seen from Figure 6a that the velocity of acoustic modes gradually decreases as the h_Metal_/λ. Among them, mode 2 exhibited the fastest change in acoustic velocity. The reflection coefficient of mode 1 dropped with the metal thin-plate thickness, while the trend of mode 2 was the opposite, as shown in Figure 6b. When h_Metal_/λ = 0.025, the reflection coefficient of mode 3 reached the minimum value. Within the calculation range, the K^2^ of all modes increased with the metal film, and finally stabilized (Figure 6c).

**Figure 7:**
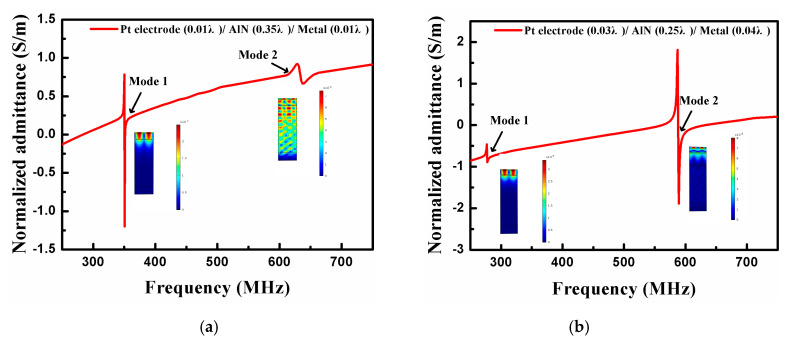

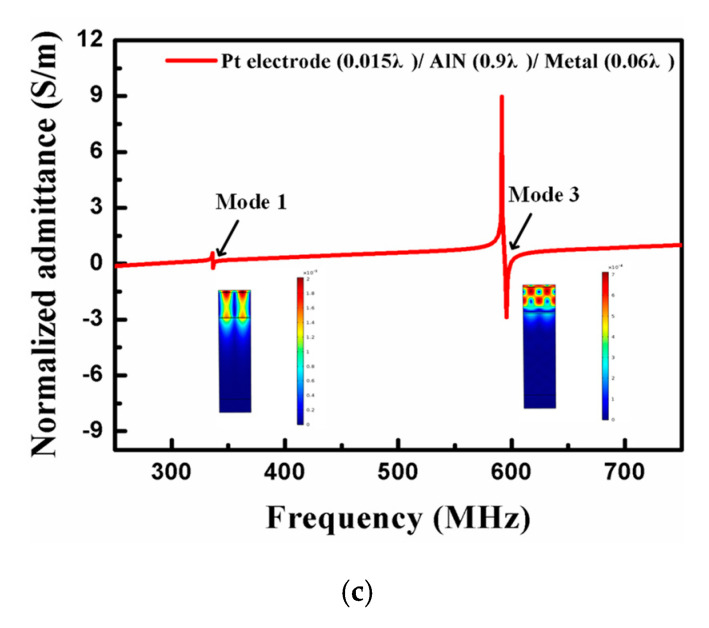
The normalized output admittance in an infinite interdigital transducers (IDT) employing optimized structure parameters when the main mode is (**a**) mode 1, (**b**) mode 2, and (**c**) mode 3.

The optimized device structure parameters can be determined by investigating the influence of different structure layers on the SAW propagation characteristics. Figure 7 exhibits the normalized output admittance in an infinite IDT with optimized structure parameters. Compared with Figure 3, it is clear that a generated primary mode accompanied by a suppressed mode can be observed. There is no denying that the number of stray modes propagating in SAW devices is reduced by optimization, making it possible to realize high-performance SAW sensors at extremely high-temperature.

## 3. Optimized Sensor Device Characterizing

**Figure 8:**
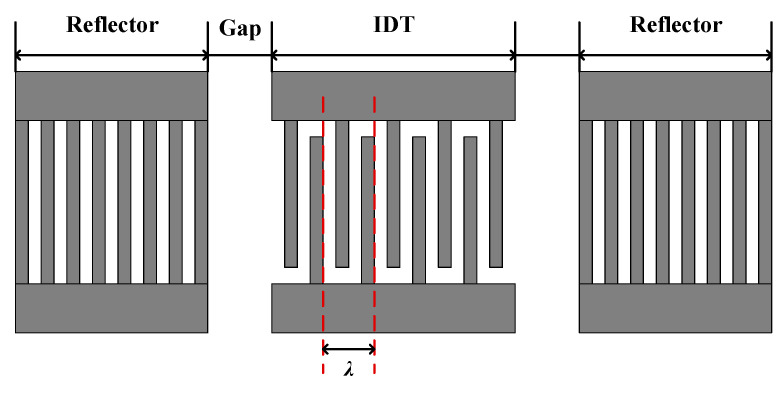
The schematic of the synchronous one-port SAW resonator.

SAW sensors generally adopt a delay line structure or a resonator structure. Compared with the delay line, the SAW resonator has high Q-value and low insertion loss [26]. Therefore, it is widely used in wireless and passive SAW sensors, especially for sensing at high temperatures. Here, the synchronous one-port SAW resonator (Figure 8) will be used for the next simulation analysis.

SAW devices can be divided into several parts with periodic or quasi-periodic structures, and each part can be represented by a P matrix. In this case, the P-matrix of IDT transducer is cascaded with the P-matrix of the reflector by the cascade relation of the mixed P-matrix, and then the P-matrix of the whole device is obtained by two cascaded matrices. Finally, based on the above calculation process, the reflection coefficient S_11_ of the proposed SAW device with the configuration of a one-port resonator can be expressed by Equation (5) [27]. The Q factor of the SAW device is estimated by Equation (6).

(5) $$P=\left[\begin{array}{ccc} P_{11} & P_{12} & P_{13}\\ P_{21} & P_{22} & P_{23}\\ P_{31} & P_{32} & P_{33} \end{array}\right],S_{11}=\frac{R-1/P_{33}}{R+1/P_{33}}.$$

(6) $$Q=\frac{f_{r}}{\Delta f_{-3\mathrm{dB}}}$$

where *R* is the equivalent source resistance and *f_r_* is the resonance frequencies.

**Figure 9:**
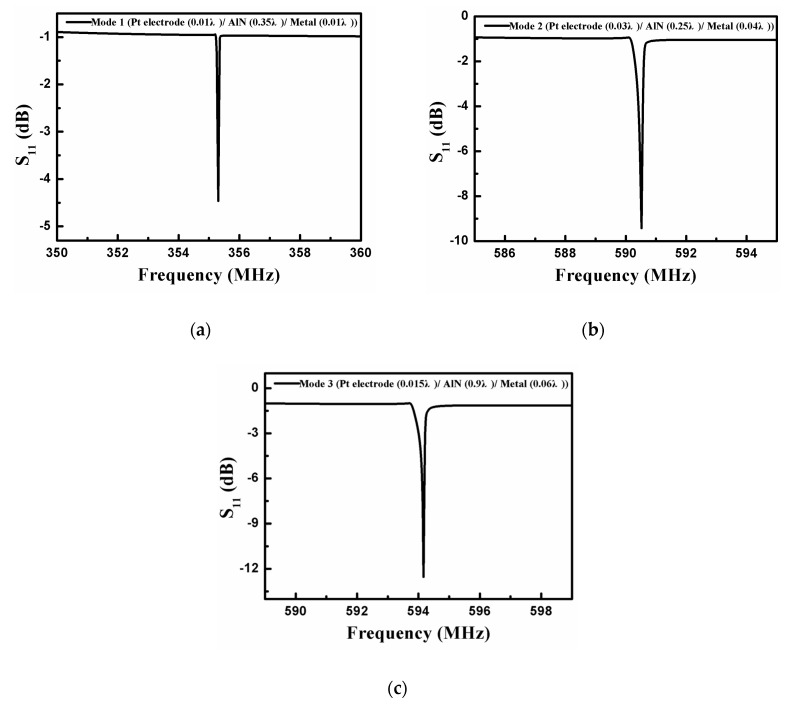
The frequency response (S_11_) of the synchronous one-port SAW resonator when the operating mode is (**a**) mode 1, (**b**) mode 2, and (**c**) mode 3.

The frequency characteristics (S_11_) of the synchronous one-port SAW resonator designed based on the above optimization results are characterized Figure 9, and the device structural parameters are shown in Table 1.

It can be seen from Figure 9 that the optimized synchronous one–port SAW resonator has excellent frequency characteristic curves. Mode 1, mode 2, and mode 3 are used as the operating modes successively, and the corresponding resonant frequencies of the optimized devices are 355.3 MHz (Figure 9a), 590.5 MHz (Figure 9b), and 594.2 MHz (Figure 9c), respectively. It is worth mentioning that there is no obvious spurious resonance except for the primary resonance in these one-port SAW resonators prepared from different acoustic modes. Meanwhile, the calculated Q factor of the SAW sensors developed in mode 1, mode 2, and mode 3 as the main mode can reach 6542, 8762, and 12,850 respectively. It shows that the optimized SAW resonator has high resonance characteristics, which is conducive to wireless sensing at extremely high temperatures.

## 4. Conclusions

In this study, a SAW device based on AlN piezoelectric film for sensing at extremely high temperature was proposed, and its basic structure is Al_2_O_3_/IDTs/AlN/metal thin-plate/Si. The generation and propagation characteristics of SAW in a multilayer composite structure were calculated systematically by the FEM analysis. Furthermore, different acoustic wave modes were optimized to meet the diversification of practical application requirements. The results show that the main propagation acoustic mode in the device is successfully excited, the spurious modes are suppressed, and the SAW device with a larger Q–value was obtained by optimizing the device. This work provides theoretical guidance for the optimization of the SAW sensor at extremely high temperatures.

## Figures and Tables

**Table 1:** The parameters of the synchronous one-port surface acoustic wave (SAW) resonator.

Mode	λ (μm)	Number of IDT Pairs	Number of Reflector Pairs	Aperture	Gap
Mode 1	13.18	120	100	100 λ	0.625 λ
Mode 2	13.18	100	80	100 λ	0.625 λ
Mode 3	13.18	100	100	100 λ	0.625 λ
